# The associations between plant-based dietary indices with depression and quality of life and insomnia among Iranian adolescent girls in 2015

**DOI:** 10.1038/s41598-024-61952-0

**Published:** 2024-05-22

**Authors:** Azam Ahmadi Vasmehjani, Zahra Darabi, Majid Ghayour-Mobarhan, Gordon A. Ferns, Sayyed Saeid Khayyatzadeh

**Affiliations:** 1 Research Center for Food Hygiene and Safety, School of Public Health Shahid Sadoughi University of Medical Sciences, Yazd, Iran; 2 Department of Nutrition, School of Public Health, Shahid Sadoughi University of Medical Sciences, Yazd, Iran; 3 Nutritional Health Research Center, Lorestan University of Medical Sciences, Khorramabad, Iran; 4 International UNESCO Center for Health-Related Basic Sciences and Human Nutrition, Mashhad University of Medical Sciences, Mashhad, Iran; 5 Division of Medical Education, Brighton & Sussex Medical School, Falmer, Brighton, Sussex BN1 9PH UK

**Keywords:** Plant-based diet index, Sleep, Depression, Quality of life, Adolescents, Diseases, Health care

## Abstract

Although previous research has explored the link between plant-based diets and mental health outcomes, there has been limited study on the quality levels of plant foods in this context. This study was conducted on 733 adolescent girls from cities in northeastern Iran. The validated Iranian version of the Insomnia Severity Index, SF-12v2 questionnaire and Persian version of the Beck Depression Inventory used to assess insomnia and poor quality of life (QoL) and depression, respectively. Dietary intakes assessed using a valid and reliable food frequency questionnaire. The association of scores of plant based dietary index (PDI) and poor QoL, depression and insomnia explored by binary logistic regression. The unadjusted model showed subjects in the highest quartile of healthy PDI had lower chances of insomnia than those in the lowest quartile (OR: 0.50; 95% CI 0.27–0.91, P = 0.024). The association persisted across various adjusted models. Subjects in the highest quartile of unhealthy PDI (uPDI) had higher chances of depression than those in the lowest quartile (OR: 1.83; 95% CI 1.09–3.08, P = 0.022). The significance of the association was maintained after adjusting for other confounders. A healthy plant-based dietary index is associated with a lower odds of insomnia. An unhealthy plant-based dietary index was associated to an increased chance of depression. Findings need to be confirmed by future studies.

## Introduction

Adolescence is a vulnerable period for mental health challenges^1^. Depression impacts between 3.2 and 8.9% of adolescents worldwide, and higher in girls than boys^2^. Depression in adolescents is linked to feelings of hopelessness, a sense of inadequacy, diminished productivity and low self-esteem^3^; therefore addressing depression and managing its symptoms in young individuals is crucial for healthcare providers and physicians. There is high prevalence of sleep disturbances among patients with depression^4^. Estimates regarding the prevalence of insomnia among adolescents vary widely, ranging from 7 to 40%^5^. Insomnia and depression are both linked to reductions in quality of life (Qol)^6^. According to the definition of the World Health Organization, QoL is "the perception of individuals about their position in life in the context of the culture and value systems in which they relate to their goals, expectations, standards and concerns"^7^.

Numerous studies have investigated the impact of plant-based diets on health outcomes, as some have reported that adherence to these dietary patterns may be associated with reduced depression rates^8,9^, insomnia^10,11^ and QoL^12^. Similarly, review studies have suggested that diets of lower quality are associated with an increased risk of depression^13^, poor QoL^14^ and sleep quality^15^. However, research on plant-based diets did not distinguish differentiate between various types of plant-derived foods including high quality plant-based such as fruits, vegetables and whole grain from low quality plant-based refined grains, potatoes and sugar sweetened beverages (SSBs)^16,17^. Findings regarding the impact of different plant-based foods on mental health have been inconsistent. Notably, a recent review study indicated that a vegetarian diet might be linked to an elevated risk of depression^18^.

Consequently, indices have been proposed for assessing dietary quality, such as the “Plant-Based Dietary Index” (PDI)^19^. Three types of PDI defined: overall PDI that included intake of all plant foods but with a decreased intake of animal foods, a healthy plant-based dietary index (hPDI) which in is concentrates on healthful plant foods, and an unhealthy plant-based dietary index (uPDI) that focuses on the consumption less-healthy plant-based foods^20^. Studies on PDI and its relation to some chronic disease are growing. Studies have indicated that greater adherence to a hPDI may contribute to a reduction in cardiovascular diseases^21^ and prevent metabolic syndrome^22^. However, there are only a few studies available and they present conflicting results regarding the relationship between plant-based dietary indices and mental health and sleep related disorders and diminished QoL. To the best of our knowledge, there is no study in this context for adolescents. Therefore, this study aimed to investigate the association of plant-based dietary indices and depression, insomnia and poor QoL in adolescent girls.

## Methods and materials

### Study population

**Figure 1:**
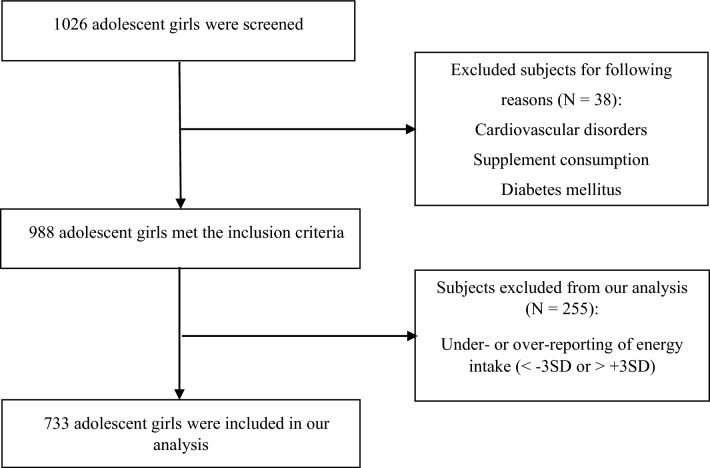
Flowchart of the data collection process of study.

The present cross-sectional study was carried out among a sample of 1026 adolescent girls (aged 12–18 years) attending 24 high schools, from six geographical areas of Mashhad and Sabzevar, located in the north-eastern Iran in 2015. Participants were selected using multi-stage cluster sampling from six geographic areas. We randomly selected four high schools from each of the geographic areas, and then randomly selected one class from each grade. Random selection of high schools, classes and students was done using of computer-generated random numbers. A total of 38 adolescents were excluded due to cardiovascular diseases, supplement consumption and diabetes mellitus. Out of 988 adolescent girls who met the inclusion criteria, 255 were subsequently excluded from the study; because they reported total energy intake out of the range of 800 to 4200 kcal/day. Therefore, data from 733 participants were included in the final statistical analysis (Fig. 1**)**. Adolescents with any autoimmune diseases, cancer, metabolic bone disease, hepatic or renal failure, cardiovascular disorders, mal absorption or thyroid, parathyroid, adrenal diseases and anorexia nervosa or bulimia were excluded. In addition, participants that consumed anti-inflammatory, anti-depressant, anti-diabetic, or anti-obesity drugs, vitamin D or calcium supplements and hormone therapy within the last 6 months were excluded. Before participating in the study, all adolescents and their parents completed written informed consent forms. The Ethics Committee of Mashhad University of Medical Sciences, Mashhad, Iran, approved this study. All methods were performed in accordance with the relevant guidelines and regulations.

### Demographic and anthropometric assessments

Data about age, smoking status, menstruation status, medical history, supplement intake, taking psychological treatment and chronic diseases were assessed through a demographic questionnaire which was administered. Trained investigators measured anthropometric variables including weight, height and waist circumference using the standard protocols. Body mass index (BMI) is computed as weight (kg) divided by the square of height (m^2^). Level of physical activity measured by the validated Modifiable Activity Questionnaire^23^. Physical activity was calculated based on metabolic equivalent task minutes per week.

### Dietary assessment

A 147-item food frequency questionnaire (FFQ) was utilized to assess dietary intake patterns^24^. The validity and reliability of the questionnaire have been documented in prior studies^25^. Participants answered questions regarding their daily, weekly, monthly and yearly consumption, as well as the frequency of food items consumed over the past year. The reported portion sizes in the FFQ were converted to grams using household measures, and the energy and nutrient intakes were calculated using the Nutritionist IV software^26^.

We created 3 versions of a plant-based diet index using dietary data: PDI; hPDI; and uPDI, as established in prior research^27^. Eighteen food groups are categorized into three main classes: healthy, unhealthy plant foods and animal foods. Healthy food groups include fruits, vegetables, whole grains, legumes, vegetable oils, nuts, tea and coffee. Less healthy food groups include sugar-sweetened beverages, refined grains, fruit juices, potato, sweets and desserts. Animal food groups include dairy products, eggs, animal fats, fish and seafood, poultry and red meat, and miscellaneous animal-based foods. These eighteen food groups were ranked into quintiles and given scores between 1 and 5. For making PDI, the highest quintile of a healthy and less healthy received a score of 5 and the lowest quintile received a score of 1. Participants in the highest quintile of animal food groups received a score of 1 and those to the lowest quintile received a score of 5. For hPDI, a positive score was considered for healthy plant food groups and a reverse score was considered for unhealthy plant food groups and animal food groups. For creating uPDI, a score of 1 was given to the lowest quintile of less healthy plant food groups and 5 for the highest quintile, whereas reverse scores were applied to a healthy plant and animal food groups. For calculating indices, 18 food group scores for an individual were summed, with a theoretical range of 18 (lowest possible score) to 90 (highest possible score).

### Assessment of psychological health

In this study Persian version of the Beck Depression Inventory (BDI) was used for the assessment of depression. This questionnaire comprises 21 items that evaluated a range of depression symptoms including feelings of guilt, feelings of hopelessness, sadness, crying, sleep disturbance, fear and loss of appetite over the past 2 weeks. The range of these scores was between 0 and 63 points. If the BDI score was < 16, persons was considered as not and they were characterized as depressed if the subject's score was > 16. The validity and reliability of BDI were assessed in previous studies^28^.

For the assessment of health-related QoL, the SF-12v2 questionnaire was used. This questionnaire is a short form of the SF-36 questionnaire and an improved version of SF-12v1^29^. The validity and reliability of this questionnaire were approved in Iran by a previous study^30^.

We used a validated Iranian version of the Insomnia Severity Index (ISI) questionnaire for the assessment of insomnia^31^. The ISI questionnaire included seven questions. Range of scores between 0 and 4 which is stratified into four categories as follows: 0 (None), 1 (Mild), 2 (Moderate), 3 (Severe) and 4 (Very Severe). A total score of ISI ranges between 0 to 28 points. If the total score of ISI was > 7, the participants were considered to have Insomnia.

### Statistical methods

Participants were divided into four groups based on quartiles of PDI; hPDI; and uPDI score. One-way-ANOVA analyses was used for comparing general characteristics and anthropometric indices of the study population across quartiles of PDI; hPDI; and uPDI scores. Univariate and multivariate regression were applied in crude and adjusted models to investigate the association quartile of PDI; hPDI; and uPDI score and poor QoL, depression and insomnia. In the adjusted models, we adjusted for age and energy intake in Model I. Additionally, adjustment was done for percentile BMI in Model II and finally, age, energy intake, BMI percentile, physical activity, menstrual status, father's job and mother's job were adjusted in the model III. All statistical analyses were performed using the SPSS v 23. P-values less than 0.05 were characterized as statistically significant.

### Ethical approval and consent to participate

The ethical committee of Mashhad University of Medical Sciences approved the written informed consent (code number: 931188). The written informed consent was signed by all participants before the beginning study.

## Results

### General characteristics study participants

The mean age of the participants was 14.5 years. The prevalence of depression, poor QoL and insomnia were 24%, 49% and 49.8% respectively. General characteristics and anthropometric indices of the participants across quartiles of PDI, hPDI, uPDI are demonstrated in Table 1. Level of age, scores of the QoL and insomnia were not different between quartiles of PDI, hPDI, uPDI. Participants in the lowest quartile of uPDI had lower depression scores compared the highest quartile. Percentile BMI and weight was significantly higher in the lowest adherence to uPDI than those in highest adherence to uPDI.

**Table 1:** General characteristics and Anthropometric indices of study participants by quartiles of PDI, uPDI, hPDI.

Variables	PDI					hPDI					u PDI				
	Q1	Q2	Q3	Q4	P value^1^	Q1	Q2	Q3	Q4	P value^1^	Q1	Q2	Q3	Q4	P value^1^
Age (year)	14.44 ± 1.55	14.48 ± 1.47	14.45 ± 1.55	14.67 ± 1.56	0.450	14.38 ± 1.52	14.66 ± 1.56	14.48 ± 1.48	14.50 ± 1.56	0.347	14.56 ± 1.50	14.37 ± 1.60	14.52 ± 1.56	14.60 ± 1.45	0.491
Percentile BMI (Kg/m2)	50.15 ± 28.77	45.65 ± 30.76	48.89 ± 28.32	46.62 ± 27.74	0.442	44.46 ± 28.23	48.55 ± 29.18	45.47 ± 27.85	52.64 ± 29.82	0.034	51.22 ± 29.82	52.0 ± 28.78	45.87 ± 28.8	42.16 ± 27.28	0.003
Weight (Kg)	53.32 ± 12.92	52.01 ± 11.91	53.00 ± 11.47	52.67 ± 10.91	0.751	51.31 ± 10.80	52.95 ± 11.28	51.47 ± 10.84	55.18 ± 13.70	0.006	54.40 ± 12.23	53.96 ± 13.03	51.90 ± 11.34	50.74 ± 10.01	0.009
Waist circumference (cm)	70.64 ± 9.69	70.18 ± 9.14	71.12 ± 8.71	69.95 ± 8.93	0.605	69.69 ± 8.69	70.55 ± 8.30	69.41 ± 8.67	72.32 ± 10.44	0.012	71.38 ± 9.32	71.35 ± 10.26	70.01 ± 8.47	69.30 ± 8.06	0.076
Metabolic equivalent task (h/week)	45.05 ± 3.34	45.09 ± 2.89	45.77 ± 3.56	45.49 ± 3.89	0.124	45.29 ± 3.14	45.98 ± 4.04	44.93 ± 3.09	45.19 ± 3.24	0.020	45.81 ± 3.69	45.25 ± 3.32	45.42 ± 3.64	45.01 ± 3.07	0.171
Score of depression	11.8 ± 9.86	10.60 ± 8.82	10.29 ± 8.92	11.18 ± 9.60	0.409	10.60 ± 8.35	11.09 ± 9.83	11.83 ± 9.89	10.16 ± 8.85	0.371	8.60 ± 8.39	11.32 ± 9.15	12.15 ± 8.93	11.48 ± 10.23	0.001
Score of quality of life	41.88 ± 7.86	41.81 ± 8.59	42.22 ± 7.67	42.33 ± 7.83	0.911	41.77 ± 7.66	41.88 ± 7.80	41.88 ± 8.01	42.76 ± 8.46	0.622	43.39 ± 8.34	42.01 ± 7.95	41.64 ± 7.50	41.26 ± 8.03	0.066
Score of insomnia	7.21 ± 5.48	7.87 ± 5.60	8.28 ± 6.32	7.80 ± 5.11	0.642	8.57 ± 5.79	8.27 ± 5.19	6.98 ± 5.78	7.11 ± 5.89	0.156	7.65 ± 5.56	7.72 ±	7.36 ± 5.16	8.49 ± 6.24	0.572

PDI: plant dietary index, uPDI: unhealthy dietary index, hPDI: healthy dietary index, BMI: body mass index, Q: Quartile, Values are means ± SD.

^1^obtained from one-way ANOVA.

### Dietary intake of study participants

The dietary intake of study participants across quartiles of PDI, hPDI, uPDI is shown in Table 2. Participants in the highest quartiles of PDI compared with the participants who were in the lowest quartile had higher intakes of energy, protein, carbohydrate, fat, whole grain, nuts, legumes, fruits, vegetables, vegetable oils, tea and coffee, potato, SSBs, fruit juice and refined grain. Intake of whole grain, tea and coffee and vegetable oil was significantly higher in subjects in the highest quartiles of hPDI compared with the participants who were in the lowest quartile. Participants in the first quartile of uPDI had a higher intake of energy, carbohydrate, fat, protein, whole grain, legumes, nuts, vegetables, vegetable oil and fruit compared with the fourth quartile.

**Table 2:** Dietary intake of study participants by quartiles of PDI, uPDI, hPDI.

Variables	PDI					hPDI					u PDI				
	Q1	Q2	Q3	Q4	P value^1^	Q1	Q2	Q3	Q4	P value^1^	Q1	Q2	Q3	Q4	P value^1^
Energy (Kcal)	2226.89 ± 776.34	2553.79 ± 745.96	2859.44 ± 801.61	3171.03 ± 704.36	< 0.001	3120.78 ± 708.85	2839. ± 789.93	2570.73 ± 813.64	2303.01 ± 787.04	< 0.001	3023.31 ± 778.81	2789.92 ± 812.17	2649.06 ± 794.55	2391.15 ± 819.95	< 0.001
Carbohydrate (gr)	288.52 ± 98.15	345.48 ± 113.06	393.09 ± 112.93	449.53 ± 106.95	< 0.001	420.04 ± 102.82	385.7 ± 121.70	356.96 ± 127.15	318.23 ± 114.15	< 0.001	396.51 ± 112.53	375.75 ± 117.07	369.20 ± 124.73	341.34 ± 129.43	< 0.001
Protein (gr)	81.32 ± 30.50	87.93 ± 30.43	97.56 ± 31.55	100.02 ± 27.74	< 0.001	107.98 ± 28.04	96.12 ± 28.09	86.94 ± 31.34	76.62 ± 28.05	< 0.001	108.34 ± 29.70	97.27 ± 29.09	87.84 ± 27.69	74.89 ± 27.88	< 0.001
Fat (gr)	88.1 ± 43.02	97.50 ± 36.31	107.32 ± 40.51	117.28 ± 40.27	< 0.001	119.68 ± 35.53	108.5 ± 40.16	95.32 ± 39.97	87.00 ± 42.00	< 0.001	119.62 ± 40.61	107.08 ± 39.11	98.41 ± 39.46	86.51 ± 39.47	< 0.001
Whole grains (gr)	35.06 ± 64.31	61.08 ± 124.09	67.34 ± 100.17	70.02 ± 106.85	0.006	38.16 ± 59.11	62.25 ± 97.97	60.32 ± 116.49	74.99 ± 124.37	0.008	81.65 ± 108.93	63.94 ± 104.84	66.51 ± 119.86	23.42 ± 52.36	< 0.001
Fruits (gr)	113.38 ± 113.41	119.95 ± 95.04	159.68 ± 132.57	197.48 ± 154.42	< 0.001	135.85 ± 116.86	153.9 ± 136.47	144.33 ± 117.51	157.19 ± 144.80	0.390	212.78 ± 149.15	154.60 ± 116.19	133.98 ± 132.86	92.28 ± 82.90	< 0.001
Vegetables (gr)	116.24 ± 99.35	141.77 ± 108.80	165.75 ± 124.61	189.12 ± 133.70	< 0.001	149.08 ± 110.57	151.7 ± 117.76	151.52 ± 116.74	163.79 ± 135.81	0.655	229.49 ± 134.23	175.88 ± 125.73	129.10 ± 91.4	82.93 ± 67.93	< 0.001
Nuts (gr)	8.41 ± 21.37	16.22 ± 27.76	16.51 ± 28.25	24.82 ± 37.75	< 0.001	15.33 ± 23.97	15.75 ± 30.92	18.31 ± 38.69	16.9 ± 23.26	0.783	22.61 ± 27.65	16.77 ± 21.73	16.16 ± 37.65	10.74 ± 28.63	0.003
Legumes (gr)	38.09 ± 28.73	54.65 ± 51.15	57.65 ± 43.97	75.27 ± 60.20	< 0.001	51.55 ± 43.22	52.70 ± 51.50	60.51 ± 50.11	62.24 ± 49.93	0.084	71.47 ± 47.66	61.33 ± 50.35	46.25 ± 40.97	48.12 ± 52.82	< 0.001
Vegetable oils (gr)	3.77 ± 6.11	5.11 ± 7.86	7.02 ± 8.40	7.96 ± 8.72	< 0.001	4.01 ± 5.04	6.66 ± 8.44	6.31 ± 8.56	7.02 ± 9.06	0.001	7.97 ± 8.38	6.34 ± 7.61	6.19 ± 8.25	3.57 ± 7.24	< 0.001
Tea & Coffee (gr)	223.21 ± 232.05	341.11 ± 328.06	397.60 ± 329.37	564.95 ± 393.00	< 0.001	310.57 ± 271.15	383.4 ± 357.64	428.70 ± 397.97	410.44 ± 339.10	0.008	397.69 ± 332.82	389.34 ± 317.56	349.94 ± 331.38	398.55 ± 402.75	0.479
Potatoes (gr)	30.50 ± 22.39	43.52 ± 48.46	50.95 ± 37.54	70.28 ± 51.67	< 0.001	67.05 ± 53.74	51.69 ± 37.90	42.79 ± 41.70	34.09 ± 33.60	< 0.001	45.95 ± 39.17	47.43 ± 40.59	47.58 ± 35.83	55.20 ± 57.13	0.185
Fruit juice (gr)	11.19 ± 34.00	22.03 ± 90.75	18.51 ± 35.25	44.01 ± 188.09	0.025	31.03 ± 55.17	19.37 ± 10.90	34.53 ± 201.89	10.90 ± 23.94	0.128	30.46 ± 92.67	17.91 ± 38.69	30.55 ± 176.74	15.70 ± 41.13	0.372
Refined grains (gr)	366.57 ± 182.29	403.84 ± 233.95	464.81 ± 254.94	491.23 ± 218.56	< 0.001	490.80 ± 185.35	461.2 ± 240.02	429.87 ± 246.84	348.74 ± 221.73	< 0.001	380.29 ± 197.88	416.39 ± 208.94	440.48 ± 222.16	496.69 ± 274.73	< 0.001
Sugar sweetened beverages (gr)	23.07 ± 65.04	40.51 ± 69.06	60.28 ± 88.17	106.69 ± 157.80	< 0.001	90.87 ± 119.22	58.30 ± 85.03	46.52 ± 91.16	35.41 ± 116.69	< 0.001	43.66 ± 106.09	46.28 ± 60.66	69.56 ± 117.46	71.12 ± 124.04	0.014
Sweet dessert (gr)	26.41 ± 22.15	37.21 ± 24.44	46.52 ± 36.61	55.63 ± 34.82	< 0.001	54.53 ± 30.65	46.44 ± 38.67	36.31 ± 25.59	29.14 ± 25.11	< 0.001	40.00 ± 37.53	43.61 ± 30.00	40.76 ± 27.07	42.67 ± 34.03	0.692
Dairy (gr)	443.83 ± 356.14	414.50 ± 276.01	431.8 ± 292.59	358.76 ± 262.38	0.040	546.00 ± 347.16	435.4 ± 276.09	364.63 ± 262.57	303.28 ± 249.01	< 0.001	573.88 ± 354.57	450.92 ± 286.65	363.58 ± 242.76	267.07 ± 209.23	< 0.001
Eggs (gr)	20.94 ± 22.07	23.88 ± 27.43	18.60 ± 15.20	18.71 ± 19.36	0.056	24.96 ± 23.08	20.89 ± 19.17	17.66 ± 19.56	18.34 ± 23.01	0.005	28.06 ± 27.99	22.92 ± 19.69	17.27 ± 17.98	13.87 ± 15.43	< 0.001
Fish or Seafood (gr)	9.09 ± 9.73	7.79 ± 10.54	9.95 ± 22.74	6.74 ± 8.17	0.154	11.97 ± 13.42	8.77 ± 9.33	8.44 ± 23.53	4.65 ± 6.40	< 0.001	12.88 ± 12.00	10.26 ± 22.55	7.26 ± 10.37	3.47 ± 6.72	< 0.001
Meat (gr)	46.20 ± 36.73	42.17 ± 31.55	48.25 ± 27.23	41.54 ± 32.19	0.176	60.18 ± 36.16	51.80 ± 40.43	38.23 ± 26.20	27.56 ± 22.15	< 0.001	56.00 ± 35.06	51.02 ± 35.36	41.60 ± 31.4	30.10 ± 31.22	< 0.001
Animal fat (gr)	11.44 ± 13.64	9.94 ± 13.74	9.02 ± 16.68	7.27 ± 14.08	0.067	12.41 ± 16.85	10.96 ± 14.49	6.98 ± 12.28	6.98 ± 14.33	< 0.001	14.42 ± 16.45	11.80 ± 17.08	7.24 ± 13.22	4.18 ± 8.51	< 0.001
Misc. animal-based foods (gr)	15.21 ± 37.88	14.24 ± 23.21	11.63 ± 18.57	12.96 ± 17.69	0.541	21.36 ± 25.51	12.62 ± 19.85	13.35 ± 36.21	6.50 ± 11.08	< 0.001	20.33 ± 25.06	13.27 ± 19.67	13.25 ± 34.10	6.85 ± 15.36	< 0.001

PDI: plant dietary index. uPDI: unhealthy dietary index. hPDI: healthy dietary index. Q: Quartile. Values are means ± SD.

^1^obtained from one-way ANOVA.

### Association between food groups and depression, poor quality of life

Multi-variable adjusted odds ratios (ORs) for depression, poor QoL and insomnia categories across quartiles of food intake are represented in Table 3. There was no association between a score of PDI and poor QoL, depression and insomnia. Subjects in the highest quartile of hPDI had lower odds of insomnia compared with the subjects in the first quartile (OR: 0.50; 95% CI 0.27–0.91, P = 0.024) in the crude model. This association remained significant after adjustments for age, energy intake, BMI percentile, physical activity, menstruation, father's job, and mother's job (OR: 0.47; 95% CI 0.24–0.93, P = 0.032). However, a score of hPDI was not significantly associated with the odds of depression and poor QoL. Highest adherence to an uPDI increased the odds of depression (OR: 1.83; 95% CI 1.09–3.08, P = 0.022). There was no association between uPDI and odds of insomnia and poor QoL.

**Table 3:** OR (95%CI) of depression , poor QoL and insomnia by quartiles of PDI, uPDI , hPDI.

			Variable	Quartiles of PDI	P value^1^	P trend
Depression	Q1	Q2	Q3	Q4		
Crude	1.00	0.68 (0.42_1.10)	0.64 (0.40_1.02)	0.70 (0.43_1.14)	0.156	0.143
Model1	1.00	0.69 (0.42_1.11)	0.65 (0.40_1.05)	0.71 (0.42_1.21)	0.218	0.204
Model2	1.00	0.68 (0.42_1.10)	0.65 (0.40_1.05)	0.71 (0.42_1.20)	0.206	0.198
Model3	1.00	0.65 (0.40_1.07)	0.64 (0.39_1.06)	0.73 (0.42_1.25)	0.257	0.263
Poor quality of life						
Crude	1.00	0.81 (0.53_1.25)	0.70 (0.46_1.06)	0.70 (0.45_1.08)	0.110	0.081
Model1	1.00	0.80 (0.52_1.23)	0.68 (0.44_1.05)	0.66 (0.41_1.06)	0.090	0.069
Model2	1.00	0.78 (0.51_1.21)	0.68 (0.44_1.05)	0.65 (0.40_1.05)	0.080	0.065
Model3	1.00	0.77 (0.49_1.20)	0.69 (0.44_1.07)	0.66 (0.41_1.08)	0.103	0.094
Insomnia						
Crude	1.00	1.44 (0.79_2.60)	1.70 (0.95_3.04)	1.55 (0.83_2.86)	0.162	0.118
Model1	1.00	1.38 (0.75_2.55)	1.65 (0.89_3.06)	1.50 (0.76_2.96)	0.234	0.176
Model2	1.00	1.39 (0.75_2.57)	1.66 (0.90_3.06)	1.50 (0.76_2.96)	0.233	0.176
Model3	1.00	1.33 (0.71_2.51)	1.50 (0.79_2.85)	1.42 (0.70_2.87)	0.322	0.281
*Quartiles of hPDI score*						
Depression	Q1	Q2	Q3	Q4		
Crude	1.00	1.25 (0.77_2.00)	1.46 (0.90_2.37)	0.98 (0.52_1.62)	0.955	0.886
Model1	1.00	1.22 (0.75_1.97)	1.40 (0.85_2.30)	0.92 (0.54_1.57)	0.767	0.892
Model2	1.00	1.23 (0.76_2.00)	1.40 (0.85_2.31)	0.94 (0.55_1.60)	0.826	0.948
Model3	1.00	1.09 (0.66_1.78)	1.40 (0.84_2.33)	0.86 (0.50_1.50)	0.614	0.848
Poor quality of life						
Crude	1.00	0.77 (0.51_1.16)	0.85 (0.55_1.29)	0.70 (0.46_1.06)	0.098	0.157
Model1	1.00	0.74 (0.49_1.29)	0.80 (0.52_1.24)	0.64 (0.41_1.01)	0.056	0.094
Model2	1.00	0.75 (0.49_1.13)	0.80 (0.52_1.24)	0.66 (0.42_1.04)	0.078	0.122
Model3	1.00	0.71 (0.46_1.08)	0.78 (0.49_1.22)	0.62 (0.39_1.00)	0.051	0.090
*Insomnia*						
Crude	1.00	1.27 (0.72_2.24)	0.74 (0.40_1.37)	0.50 (0.27_0.91)	0.024	0.008
Model1	1.00	1.26 (0.70_2.28)	0.74 (0.39_1.40)	0.50 (0.26_0.95)	0.037	0.012
Model2	1.00	1.26 (0.70_2.27)	0.74 (0.39_1.40)	0.49 (0.25_0.94)	0.034	0.012
Model3	1.00	1.24 (0.66_2.30)	0.73 (0.37_1.42)	0.47 (0.24_0.93)	0.032	0.010
*Quartiles of uPDI score*						
Depression	Q1	Q2	Q3	Q4		
Crude	1.00	1.60 (0.95_2.70)	2.34 (1.42_3.86)	1.83 (1.09_3.08)	0.022	0.009
Model1	1.00	1.60 (0.94_2.70)	2.33 (1.40_3.87)	1.82 (1.06_3.11)	0.028	0.012
Model2	1.00	1.79 (1.04_3.07)	2.31 (1.39_3.84)	1.79 (1.04_3.07)	0.034	0.015
Model3	1.00	1.61 (0.94_2.75)	2.28 (1.35_3.84)	1.76 (1.00_3.09)	0.048	0.024
Poor quality of life						
Crude	1.00	1.54 (1.01_2.34)	1.69 (1.12_2.56)	1.71 (1.12_2.62)	0.012	0.012
Model1	1.00	1.57 (1.03_2.39)	1.72 (1.13_2.61)	1.74 (1.12_2.71)	0.013	0.013
Model2	1.00	1.57 (1.03_2.40)	1.68 (1.10_2.57)	1.69 (1.08_2.63)	0.020	0.021
Model3	1.00	1.57 (1.02_2.43)	1.62 (1.05_2.51)	1.56 (0.98_2.47)	0.060	0.067
Insomnia						
Crude	1.00	0.88 (0.48_1.63)	0.69 (0.38_1.26)	1.11 (0.61_2.02)	0.730	0.923
Model1	1.00	0.99 (0.52_1.87)	0.73 (0.40_1.36)	1.27 (0.67_2.46)	0.443	0.672
Model2	1.00	1.00 (0.53_1.88)	0.74 (0.40_1.38)	1.30 (0.67_2.51)	0.425	0.651
Model3	1.00	0.99 (0.51_1.93)	0.76 (0.40_1.44)	1.35 (0.64_2.71)	0.397	0.602

PDI: plant dietary index. uPDI: unhealthy dietary index . hPDI: healthy dietary index.^1^Fourth quartile compared to first quartile

Model 1: Adjusted for age and energy intake.

Model 2: additionally, adjusted for BMI percentile.

Model 3: additionally, adjusted for physical activity, menstruation, father's job, mother's job.

## Discussion

The present study suggests that a higher hPDI score is associated with a reduced chance of insomnia. High scores on the uPDI was associated with an increased risk of depression. There is limited cross-sectional evidence on the association between PDI scores and sleep quality, mental health profile and poor QoL. In agreement with us, Haghighatdoost et al.^32^ found that higher compliance with an uPDI was associated with a higher risk of depression whereas PDI and hPDI were not related to it, As Daneshzad et al.^33^ and zamani et al.^34^, both reported that a high adherence of an uPDI increased risk of depression in diabetic women and healthy, respectively. Some potential explanations for the correlation between a low PDI score and an increased risk of depression could be attributed to the dietary intakes of the participants in this study. So that, those in the upper quartile had lower consumption of healthful plant foods such as whole grains, legumes, nuts, vegetables, vegetable oils and fruits, and higher consumption of unhealthy plant foods such as refined grains and SSBs, which may influence on inflammation and antioxidant levels, potentially influencing brain function^35,36^. Additionally, a potential contributing factor to the risk of depression could be the deficiency of nutrients like vitamin D and B12, typically found in animal products, while participants with higher uPDI scores in this study, reported significantly lower consumption of these types of foods, such as dairy products, eggs, fish or seafood, meat, animal fats and other animal-based foods^37,38^. However, a large cross-sectional study involving Iranian adults observed no association between the uPDI and the odds of depression^39^. Differences in findings may stem from the varied methodologies used to assess depression and dietary intake.

Our findings showed no significant link between overall PDI, hPDI, and uPDI scores and poor QoL after adjusting for all confounding factors. No study has yet evaluated the relationship between PDI indices and QoL. However, a cross-sectional, multicentric study aligns with our findings, indicating that a higher uPDI is associated with an increased risk of depression, whereas PDI and hPDI show no association with depression^32^. In this line, the Nurses' Health Study demonstrated that a decline in the quality of a plant-based diet was correlated with a lower QoL^40^. A possible explanation based on previous studies may be an increased depression due to a lower intake of antioxidant vitamins and fibers from fruits and vegetables^41,42^ which may influence on QoL However, the mechanisms by which dietary quality directly influences QoL are still not fully understood; therefore, specific studies are required to assess the relationship between plant-based diet indices and QoL.

Recent research indicates a connection between diet and sleep quality. A systematic review ^15^ has revealed that a diet rich in healthy foods correlates with improved sleep quality, whereas a diet high in processed and sugar-rich foods s associated with poorer sleep patterns. Data from a cohort study^43^ suggested that individuals with poor quality of sleep consumed fewer healthful plant-based foods including fruits, vegetables, whole grains and legumes. In our population a higher adherence of the hPDI was correlated to lower odds insomnia. Consistent with our findings, Ferranti et al. reported a positive association between fruits and vegetables intake and sleep duration^44^. A healthful plant-based diet that includes foods rich in vitamins, minerals, antioxidants, phytochemicals may through to stop inflammation and to reduce oxidative stress, are related to sleep disturbances^45^. Phytochemicals found in healthful plant-based foods, such as flavonoids predominantly present in (fruits, vegetables, tea, coffee) and phenolic acids in (fruits, coffee, pulses, nuts) and phytoestrogens like isoflavones and lignans in (legumes), may be associated with improved sleep quality among individuals^45^. Numerous studies have demonstrated that certain flavonoids, such as apigenin and hydroxycinnamic acids, enhance cellular antioxidant defense against oxidative stress in the central nervous system^46^. Moreover, lignans have been shown to exert anti-oxidative and anti-inflammatory effects on neurons, as well as protect the blood–brain barrier from inflammatory cells by mitigating oxidative stress, inflammation, and permeability^47^. Additionally, melatonin found in legumes^48^ and fruits and vegetables^49^ may help alleviate sleep disorders. St-Onge et al.^50^ and lin et al.^51^, has indicated that fruits such as tart cherries, rich in melatonin and phytonutrients, along with kiwifruit, abundant in folate and serotonin, can potentially enhance sleep quality. Furthermore, antioxidant vitamins like vitamin C and E may bolster sleep by protecting against damaging free radicals^51^. It has been suggested that a diet high in protein could boost alertness by increasing tyrosine levels, an amino acid, and by stimulating the production of catecholamines^52,53^. This contrasts with our study population, where individuals with higher adherence to the hPDI reported lower protein consumption. Previous reviews have indicated that consuming foods rich in tryptophan, may be associated with enhanced sleep quality^54^ A study has reported that the intake of tryptophan is positively correlated with sleep duration^55^. However, some research has not established a connection between tryptophan-rich healthy plant-based diets and sleep quality. This lack of association could be attributed to the suboptimal tryptophan dosage for participants, changes in sleep patterns over time, and the impact of meal timing and body composition on sleep^56,57^.

This study is the first to explore the association between plant-based diet indices and conditions such as insomnia, depression, and poor QoL in Iranian adolescent girls. We used a valid and reliable FFQ to assess dietary intakes. However, this study had some limitations, such as the cross-sectional design that cannot determine causality and therefore, it required prospective studies to verify findings. Potential misclassification may occur due to measurement errors in FFQ. Our study did not account for hormonal changes as a potential confounding factor affecting mood during menstruation in girls. Additionally, it should be noted that our study population was confined to adolescent girls.

## Conclusions

We have shown that higher adherence to an unhealthy plant-based diet correlates with an increased risk of depression, whereas a stronger compliance to healthy plant foods is linked to a reduced risk of insomnia. However, no significant relevance was found between PDI, hPDI, and uPDI scores and poor QoL. Nonetheless, our findings require validation through longitudinal or follow-up studies.

## Data Availability

The data and materials of the present study are available from the corresponding author on reasonable request.

## Abbreviations

BDI: Beck depression inventory
BMI: Body mass index
PDI: Plant-based diet index
hPDI: Healthful PDI
uPDI: Unhealthy PDI
FFQ: Food frequency questionnaire
MAQ: Modifiable activity questionnaire
OR: Odds ratio
Qol: Quality of life
MET: Metabolic equivalent task
SD: Standard deviation
SPSS: Statistical package for social science
WC: Waist circumference
